# Mammographic density and breast cancer risk in breast screening assessment cases and women with a family history of breast cancer

**DOI:** 10.1016/j.ejca.2017.10.022

**Published:** 2018-01

**Authors:** Stephen W. Duffy, Oliver W.E. Morrish, Prue C. Allgood, Richard Black, Maureen G.C. Gillan, Paula Willsher, Julie Cooke, Karen A. Duncan, Michael J. Michell, Hilary M. Dobson, Roberta Maroni, Yit Y. Lim, Hema N. Purushothaman, Tamara Suaris, Susan M. Astley, Kenneth C. Young, Lorraine Tucker, Fiona J. Gilbert

**Affiliations:** a Centre for Cancer Prevention, Wolfson Institute of Preventive Medicine, Charterhouse Square, London EC1M 6BQ, UK; b Department of Medical Physics and Clinical Engineering, Cambridge University Hospitals NHS Foundation Trust, Addenbrooke's Hospital, Cambridge Biomedical Campus, Hills Road, Cambridge CB2 0QQ, UK; c Aberdeen Biomedical Imaging Centre, Lilian Sutton Building, Foresterhill, University of Aberdeen, Aberdeen AB25 2ZD, UK; d Department of Radiology, Cambridge University Hospitals NHS Foundation Trust, Addenbrooke's Hospital, Cambridge Biomedical Campus, Hills Road, Cambridge CB2 0QQ, UK; e Jarvis Breast Centre, 60 Soughton Road, Guildford GU1 1LJ, UK; f North-East Scotland Breast Screening Centre, Foresterhill Road, Foresterhill, Aberdeen AB25 2XF, UK; g Breast Radiology Department, King's College Hospital NHS Foundation Trust, Denmark Hill, London SE5 9RS, UK; h West of Scotland Breast Screening Service, Stock Exchange Court, 77 Mandela Place, Glasgow G2 1QT, UK; i The Nightingale Centre & Genesis Prevention Centre, University Hospital of South Manchester, Southmoor Road, Manchester M23 9LT, UK; j Imperial College Healthcare NHS Trust, Fulham Palace Road, London W6 8RF, UK; k Breast Screening Unit, St Bartholomew's Hospital, London EC1A 7BE, UK; l Centre for Imaging Sciences, Institute of Population Health, University of Manchester, Oxford Road, Manchester M13 9PT, UK; m National Co-ordinating Centre for Physics of Mammography, Royal Surrey County Hospital, Guildford GU2 7XX, UK

**Keywords:** Breast cancer screening, Breast cancer risk, Mammographic density, Automated volumetric method, Quantra, Volpara, Digital breast tomosynthesis, Fibroglandular volume

## Abstract

**Background:**

Mammographic density has been shown to be a strong independent predictor of breast cancer and a causative factor in reducing the sensitivity of mammography. There remain questions as to the use of mammographic density information in the context of screening and risk management, and of the association with cancer in populations known to be at increased risk of breast cancer.

**Aim:**

To assess the association of breast density with presence of cancer by measuring mammographic density visually as a percentage, and with two automated volumetric methods, Quantra™ and VolparaDensity™.

**Methods:**

The TOMosynthesis with digital MammographY (TOMMY) study of digital breast tomosynthesis in the Breast Screening Programme of the National Health Service (NHS) of the United Kingdom (UK) included 6020 breast screening assessment cases (of whom 1158 had breast cancer) and 1040 screened women with a family history of breast cancer (of whom two had breast cancer). We assessed the association of each measure with breast cancer risk in these populations at enhanced risk, using logistic regression adjusted for age and total breast volume as a surrogate for body mass index (BMI).

**Results:**

All density measures showed a positive association with presence of cancer and all declined with age. The strongest effect was seen with Volpara absolute density, with a significant 3% (95% CI 1–5%) increase in risk per 10 cm^3^ of dense tissue. The effect of Volpara volumetric density on risk was stronger for large and grade 3 tumours.

**Conclusions:**

Automated absolute breast density is a predictor of breast cancer risk in populations at enhanced risk due to either positive mammographic findings or family history. In the screening context, density could be a trigger for more intensive imaging.

## Highlights

• Three different breast density measures compared: visual, Quantra and Volpara.
• All density measures showed a positive association with presence of cancer.
• The strongest effect was seen with Volpara absolute density measure.
• A stronger effect of Volpara density on risk was observed for large and grade 3 cancers.

## 1 Introduction

High breast density has been shown to be a strong, independent risk factor for breast cancer [1], [2], [3], [4], [5]. It has been reported that women with a high breast density compared to women with a low breast density have a four- to sixfold increased risk of developing the disease [6], [7], [8], [9], [10]. High breast density has also been linked to cancers which are larger and have positive lymph nodes, although the reported results vary considerably [11], [12], [13], [14], [15] and high breast density has been found in women with cancers diagnosed outside of the screening programme [1], [4], [16], [17], [18]. One possible explanation for the latter is a masking bias, in that dense breast tissue could render breast cancers less sensitive to screen detection, leading to a higher incidence of breast cancer in those previously screened negative. A number of studies, however, indicate that this is only partly responsible for the observed increased cancer risk with high density [2], [6], [19]. Indeed, density has been shown to be a risk factor for screen-detected as well as symptomatic cancers [4], [6].

There is no consensus on the most useful measure of breast composition in risk prediction, risk management and surveillance decisions. One meta-analysis found that absolute rather than proportional estimates of breast density are more strongly predictive of risk [2], whereas another found the opposite [20].

Younger, pre- or perimenopausal women are known to have a higher proportion of dense breast tissue, as breast density decreases with age [21], [22]. The National Health Service Breast Screening Programme (NHSBSP) in the United Kingdom (UK) invites women aged 50–70 every 3 years for two-view digital mammography which is double read [23]. Extension of the age range to 47–73 is currently under investigation. Women at moderate risk with a significant family history of breast cancer may be screened annually from age 40 [24].

Issues outstanding in breast density include:

• identifying the breast density measure (percent density, absolute quantity of dense tissue) most strongly associated with breast cancer;
• the method of measurement (visual, automated volumetric measures, automated area measures) most strongly associated with cancer;
• age and tumour-specific associations with risk;
• the extent to which density contributes risk information in subjects already known to be at higher risk of breast cancer, such as women attending for screening who are recalled for assessment due to a suspicious mammographic finding (and which measure of density is most suitable in this population).

Also, it is worth noting that the identification of mammographic density as a risk factor took place in the predigital era, and most of the studies demonstrating the effect of density on breast cancer risk pertain to measures from film/screen mammography. There is a current need to demonstrate and validate measures of breast composition from digital mammography which are equally strongly associated with breast cancer risk.

In this study, we assess the associations of visual percent density assessment and automated volumetric breast composition measures with breast cancer risk in women recalled for assessment in the general population screening programme and in women aged 40–50 years under increased mammographic surveillance due to a family history of breast cancer. Women in the latter category are those at moderate or high familial risk of breast cancer, defined as a lifetime risk of at least 17% [24].

## 2 Materials and methods

In the TOMMY trial (TOMosynthesis with digital MammographY in the UK NHS Breast Screening Programme), participants were recruited from six centres [25]. They comprised women aged 47–73 years recalled to an assessment clinic and also women below 50 years of age with a family history of breast cancer who attended annual mammography screening. Data were available for 6020 breast screening assessment cases (of whom 1158 had breast cancer) and 1040 family history screenees (of whom two had breast cancer), who had been recruited between February 2011 and August 2013. On recruitment, each woman had a two-dimensional (2D) mammogram as part of the digital breast tomosynthesis (DBT) examination. Both the DBT and the standard 2D imaging were performed as a single procedure at the same breast compression on a Hologic Selenia Dimensions Digital Mammography Unit (Hologic Inc., Bedford, MA, United States of America ). These research images were read by trained radiologists blinded to the knowledge of cancer status of the woman-screenee, using full field digital mammography (2D) and the DBT. To score visual density, readers used a visual analogue scale (VAS), requiring them to make a mark on a 10-cm line which was subsequently converted to a percentage score between 0% and 100% [26]. Visual percent density was estimated for each woman by one of 26 image readers using information from the available mammograms from the examination without knowledge of cancer status (although the readers were of course able to see abnormalities). In the family history cases, density was also scored by an additional reader and the mean of the two results was used. Although visual assessment of density is subject to inter- and intraobserver variability [5], [27], reasonable agreement was observed between the readers, with absolute differences of less than 10% in 70% of cases [28]. The readers had a minimum of two years' experience of reading at least 5000 cases annually in the NHSBSP.

In addition to radiological, clinical and pathological data, we also measured breast density using two automated volumetric tools, Volpara™ version 1.4.2 [29] and Quantra™ version 2.0 [30] and by visual assessment. All breast density measures were performed on 2D mammography.

Age was coded for 6985 (99%) of the 7060 cases. Ages of the subjects ranged from 29 to 85, with 96% of subjects aged 40–70. Volpara breast composition data were available for 7019 of the 7060 cases (1157 of the 1160 cancer patients and 5862 non-cancer patients). Corresponding Quantra data were available for 7005 of the cases (1156 cancer patients and 5849 non-cancer patients). Visually assessed percent density was available for 6969 cases (including 1153 cancer patients). None of the three methods gave a complete set of results for all cases as the software tools did not produce scores for every image analysed and other clinical pressures occasionally took precedence over the requirement to give a density score. However, this occurred in only 0.6% and 0.8% of cases in Volpara and Quantra respectively.

The output of both software tools gave measurements of total breast volume, dense fibroglandular volume and percent volumetric breast density for each image. The craniocaudal (CC) and the mediolateral-oblique (MLO) images of each breast were analysed. To obtain a single score for each woman, the CC and MLO scores were utilised as follows. For cases where no cancer was assessed as being present, the largest breast volume and fibroglandular volume for each breast (either from the CC or MLO view) were determined and the average of each of these volumes of the two breasts were calculated. For cases where cancer was confirmed, results were used from the contralateral breast. If no contralateral data were available, results from the affected breast were used. This occurred for one cancer case in the Quantra data (0.1% of cancers) and 14 cases in the Volpara (1% of cancers). Volumetric percent density was calculated, as 100 times the ratio of the fibroglandular tissue volume to the overall breast volume.

To evaluate the association of breast composition measures with risk, data were analysed by logistic regression with breast cancer as the outcome variable and the various density and volume measures as predictor variables, adjusted for age. A major negative confounder of area or volumetric percent density is body mass index (BMI). In the NHSBSP, weight and height are not traditionally recorded. We had, however, weight and height data for a small subset of 178 recruits for which we calculated BMI. While this did not provide sufficient data to adjust the regression models, we analysed this subset and found that:

(1) BMI and total breast volume as measured by Volpara had very similar negative correlations with percent density measures; and
(2) within this subset of the data, adjusting the effects of percent density measures on breast cancer risk for total breast volume gave almost identical results to adjusting for BMI.

See Appendix for detailed numerical results.

We then compared the predictive potential of the measures using standardised logistic regression coefficients, so that all measures pertained to the same scale. Using the most predictive measure, we then estimated effects in subgroups of age, invasive status, node status, size and grade of the cancers diagnosed, all determined histologically, radiological features (mass, calcification, or either asymmetry or architectural distortion, as determined by the readers), and detection status by 2D mammography and DBT. Data were analysed using STATA version 10.0 [31].

## 3 Results

Table 1 shows the mean and standard deviation of breast composition measures using Volpara, by age, cancer status and non-cancer source (assessment or family history screenee). The dense tissue volume was generally higher in cancer cases than in non-cancer cases, and declined with age in all groups. The percent density showed the same tendencies, although less markedly. Table 2 shows the corresponding figures for Quantra, exhibiting a similar pattern. Table 3 shows the mean and standard deviation of visually assessed percent density by age, cancer status and non-cancer source. This showed a distinct decline with age for both cancer and non-cancer cases. However, in those aged 60 or over, the cancer cases had a slightly lower percent density than the non-cancer cases.

**Table 1:** Means (SD's) of breast composition measures using the Volpara volumetric breast density measurement (Volpara Health Technologies Ltd), by age and diagnostic group, in 6944 assessment cases, including 1149 cancers in the UK Breast Screening Programme.

Age (years)	Breast composition measure	Mean (SD) for population			
		Cancers	Assessment non-cancers	Family history non-cancers	All non-cancers
<50	Breast volume (cm^3^)	1063 (663)	1027 (673)	1041 (668)	1037 (669)
	Dense volume (cm^3^)	111 (53)	101 (67)	101 (61)	101 (63)
	% Density	13 (7)	12 (6)	12 (7)	12 (7)
	No. of subjects	29	313	942	1255
50–59	Breast volume (cm^3^)	1153 (670)	1034 (614)	983 (597)	1033 (613)
	Dense volume (cm^3^)	94 (54)	84 (50)	84 (51)	84 (50)
	% Density	10 (6)	9 (5)	10 (5)	10 (5)
	No. of subjects	460	3092	43	3135
≥60	Breast volume (cm^3^)	1092 (562)	1010 (557)	582 (75)	1009 (556)
	Dense volume (cm^3^)	77 (44)	73 (43)	53 (20)	73 (43)
	% Density	8 (4)	8 (4)	9 (2)	8 (4)
	No. of subjects	660	1402	3	1405

**Table 2:** Means (SD's) of breast composition measures using Quantra volumetric breast density measurement (Hologic), by age and diagnostic group, in 6930 assessment cases, including 1148 cancers in the UK Breast Screening Programme.

Age (years)	Breast composition measure	Mean (SD) for population			
		Cancers	Assessment non-cancers	Family history non-cancers	All non-cancers
<50	Breast volume (cm^3^)	1118 (723)	1075 (696)	1088 (681)	1085 (685)
	Dense volume (cm^3^)	143 (99)	142 (128)	137 (99)	138 (107)
	% Density	14 (7)	14 (7)	14 (7)	14 (7)
	No. of subjects	29	313	938	1251
50–59	Breast volume (cm^3^)	1195 (670)	1079 (636)	1044 (602)	1078 (636)
	Dense volume (cm^3^)	131 (93)	114 (99)	111 (82)	114 (88)
	% Density	12 (6)	11 (6)	13 (9)	11 (6)
	No. of subjects	459	3088	43	3131
≥60	Breast volume (cm^3^)	1126 (582)	1054 (570)	612 (121)	1052 (570)
	Dense volume (cm^3^)	104 (71)	97 (76)	57 (41)	97 (76)
	% Density	9 (5)	9 (5)	9 (5)	9 (5)
	No. of subjects	660	1397	3	1400

**Table 3:** Means (SD's) of visually assessed percent density, by age and diagnostic group, in 6969 assessment cases, including 1153 cancers in the UK Breast Screening Programme.

Age (years)	Quantity	Mean (SD) for population			
		Cancers	Assessment non-cancers	Family history non-cancers	All non-cancers
<50	% Density	46 (19)	43 (18)	42 (22)	42 (21)
	No. of subjects	29	314	915	1229
50–59	% Density	42 (22)	40 (21)	37 (18)	40 (21)
	No. of subjects	461	3067	41	3108
≥60	% Density	33 (19)	35 (20)	40 (22)	35 (20)
	No. of subjects	663	1432	47	1479

Table 4 shows the age-adjusted standardised logistic regression coefficients for the automated measures of dense tissue volume and the visually assessed percent density. The three measures that used percentages were also adjusted for Volpara total breast volume. The strongest effect in terms of both coefficient and significance was that of Volpara absolute dense tissue volume, corresponding to a 3% increase in the odds of cancer per additional 10 cm^3^ of dense tissue (95% CI 1–5%). The effect of Quantra dense tissue volume was slightly weaker but very similar. The confidence intervals on the two standardised estimates indicate that the difference is compatible with chance.

**Table 4:** Age-adjusted standardised logistic regression coefficients for effects of Volpara (Volpara Health Technologies Ltd), Quantra (Hologic) and visually assessed breast composition measures on risk of breast cancer in approximately 6900 (varying depending on numbers with missing data) assessment cases in the UK Breast Screening Programme.

Breast composition measure		Standardised logistic regression coefficient	95% CI	Exact significance
Volpara	Absolute dense volume	0.16	0.09–0.22	p = 0.000002
	Percent dense volume	0.09	0.00–0.17	p = 0.04
Quantra	Absolute dense volume	0.15	0.09–0.22	p = 0.000003
	Percent dense volume	0.14	0.06–0.21	p = 0.0003
Visual	Percent dense area	0.09	0.01–0.16	p = 0.02

Table 5 shows the age-adjusted odds ratios by quintile of the two measures of dense tissue volume, both showing a moderate but highly significant increase in risk across quintiles. There was an approximate doubling of risk for the highest quintile compared to the lowest.

**Table 5:** Odds ratios for breast cancer by quintile of absolute dense volume by Volpara (Volpara Health Technologies Ltd) and Quantra (Hologic) in approximately 6900 (depending on numbers with missing data) assessment cases in the UK Breast Screening Programme.

Volpara dense volume (cm^3^)	OR	95% CI	Quantra dense volume (cm^3^)	OR	95% CI
<48	1.00	–	<54	1.00	–
48–63.99	1.45	1.17–1.79	54–78.99	1.46	1.17–1.82
64–82.99	1.53	1.23–1.90	79–110.99	1.65	1.32–2.05
83–114.99	1.50	1.19–1.86	111–159.99	1.63	1.30–2.04
≥115	1.88	1.50–2.35	≥160	2.06	1.65–2.57

Table 6 shows the results of subgroup analyses of the association of Volpara dense tissue volume with breast cancer risk. For the most part, the effect of the volume of dense tissue was similar in subgroups to that overall, but a number of observations arise. The increased risk with this measure was for the most part apparent within the subgroups considered. The effect was slightly higher in the presence of calcifications, in tumours missed by 2D mammography, in node positive tumours, in larger tumours (>20 mm, and to a lesser extent in tumours of size 11–20 mm) and in grade 3 cancers. For the radiological indications, the effect of density on risk of tumours appearing as calcifications was statistically significant, and for tumours appearing as either asymmetry or architectural distortion the effect was of borderline significance.

**Table 6:** Odds ratios per 10 cm^3^ of dense tissue as measured by Volpara (Volpara Health Technologies Ltd) within subgroups of 7019 assessment cases in the UK Breast Screening Programme.

Variable	Subgroup	OR per 10 cm^3^	95% CI	Significance
Age (years)	<50	1.02	0.97–1.07	p = 0.3
	50–59	1.04	1.02–1.07	p < 0.001
	≥60	1.02	1.00–1.05	p = 0.03
Dominant radiological feature	Mass	1.00	0.96–1.04	p = 0.8
	Calcification	1.04	1.02–1.07	p < 0.001
	Asymmetry/architectural distortion	1.03	1.00–1.06	p = 0.03
Recalled by 2D mammography	No	1.04	1.00–1.07	p = 0.01
	Yes	1.03	1.01–1.05	p < 0.001
Recalled by 2D + DBT	No	1.01	0.96–1.06	p = 0.5
	Yes	1.03	1.01–1.05	p = 0.001
Recalled by synthetic 2D + DBT	No	1.02	0.98–1.06	p = 0.4
	Yes	1.03	1.00–1.05	p = 0.002
Invasive status	Non-invasive	1.03	1.00–1.06	p = 0.01
	Invasive	1.03	1.01–1.05	p < 0.001
Node status of tumour (invasive only)	Negative	1.02	1.00–1.05	p = 0.01
	Positive	1.03	1.01–1.06	p < 0.001
Size of tumour (invasive only)	1–10 mm	1.01	0.98–1.04	p = 0.6
	11–20 mm	1.03	1.00–1.05	p = 0.004
	>20 mm	1.06	1.03–1.08	p < 0.001
Histological grade of tumour (invasive only)	1	1.03	1.00–1.06	p = 0.01
	2	1.02	1.00–1.05	p = 0.02
	3	1.04	1.00–1.08	p = 0.001
Total population	–	1.03	1.01–1.05	p < 0.001

*Notes* Asymmetry/architectural distortion: forms of asymmetrical breast density visible from the mammogram.

## 4 Discussion

We found that automated volumetric measures of mammographic density added significantly to estimation of breast cancer risk in subjects already known to be at enhanced risk due to a screening finding or to family history. This adds to the evidence of breast density as a robust predictor of breast cancer risk. Notably, we found that the automated absolute measures were more strongly predictive of risk in this population than visually assessed percent density. The fact that automated measures were predictive indicates that density may have a role in risk management at population level. The NHSBSP screens more than two million women per year and, clearly to be practicable, any breast composition risk marker would have to be automatically derived with minimal human resource implications. Both commercially available products, Volpara and Quantra, showed predictive potential, with Volpara slightly stronger. Our risk gradients were not as strong as observed by others [32]. This may be due to the fact that our non-cancer cases were at enhanced risk due to recall for assessment or family history and therefore may have had higher breast density than general population controls. Also, our study data did not include interval cancers. There are a higher proportion of interval cancers in dense breasts, and if these had been included this would have likely increased the risk gradient to the expected level. In a single Dutch screening centre of women in the 50–75 year old category, including interval cancers, the highest quartile of absolute density had a 2.5-fold risk compared to the lowest quartile [33].

We did not have data on BMI, except for a small minority of cancers, so could not adjust for this in our analysis. However, as reported in the Appendix, in the subset with BMI data, total breast volume as assessed by Volpara displayed the same properties as BMI in terms of correlation with other breast composition measures and of adjustment of percent density measures. This raises an interesting issue. Traditionally, estimates of the effect of percent mammographic density on breast cancer risk are adjusted for BMI as the two are known to be strongly negatively confounded. The reason for this confounding may be the structural negative relationship between percent density and total breast size, since the latter is essentially the denominator of the former. Thus, BMI may be a surrogate for total breast volume rather than the reverse. In any case, results in the Appendix suggest that adjustment for total breast volume achieves the same effect in this context as adjustment for BMI.

The finding that breast density is associated with increased risk of breast cancer in this specific population already known to be at enhanced risk is novel, but consistent with the literature. While studies vary in their findings as to which measure of density is most predictive of risk, the finding that increased levels of density are associated with increased risk of breast cancer is almost universal [1], [2], [3], [4], [5], [6], [7], [8], [9], [10], [11], [12]. It has generally been observed that quantitative measures of density are stronger predictors of breast cancer risk than qualitative [2], [9]. It is known that density also impairs mammographic accuracy, which can mean that some tumours are missed at screening due to masking by high levels of density, and therefore subsequent incidence in this group is increased [13]. However, results from several studies indicate that there is also an effect of increased risk of breast cancer due to density which is not attributable to a masking phenomenon [2], [4], [6], [19]. Recent results suggest that absolute measures of dense tissue area or volume have greater predictive power than percentage measures [34], [35], [36], but this is not universally observed [37].

In this study, we found that absolute dense tissue volume was a stronger predictor than percent density. We also found that dense tissue volume was slightly more strongly predictive of cancers with unfavourable prognostic factors such as larger than 20 mm in size, grade 3 and node positive cancers. Confidence intervals were relatively wide in these subgroups, so interpretation should be done cautiously. Whether this is due to chance, to a true difference in the biological effect of high levels of density or to the masking effect of dense tissue remains to be seen, but it has been observed elsewhere [11], [12], [13], [14], [38], [39]. Again, inclusion of interval cancers (not possible in this study) would clarify issues of masking. A number of other studies, however, have not found a stronger association with less favourable biological tumour attributes [40], [41], [42], [43]. Two of the latter studies did find an increased effect of density on interval cancers which would be expected to be larger and more likely to be node positive [41], [43]. The result may be particular to screen-detected cancers, since Ding *et al.* [41] did find an increased effect of density on risk of larger tumours among their screen-detected cancers. This may have management or diagnostic implications. In our set of screen-detected cancers, larger, node positive cancers were found in the breasts with highest density. Some of these may have been missed at previous screens as a result of high density and had diagnosis considerably delayed. The UK breast screening programme has a relatively long three yearly screening interval. Therefore, screening frequency could be increased for women with more dense fibroglandular tissue in order to find the tumours at a smaller size, or DBT could be used in those women with highest breast volumetric density, as the addition of DBT was found to improve diagnostic accuracy in women with dense breasts in the TOMMY trial [25]. The adequacy of screening frequency depending on breast density (and possibly on other risk factors) is being currently studied by others, such as the PROCAS study [44].

It is worth noting that women with dense breasts were more likely to undergo biopsy. In the lowest quintile of Volpara absolute dense volume, 33% of the assessment cases had a biopsy, whereas in the highest quintile, the figure was 41%. It is possible that the availability of DBT (and perhaps other imaging modalities) at assessment might avoid some unnecessary biopsies. Of the non-cancer cases who underwent biopsy, 39% were not marked for recall in rereading by DBT (compared to 29% in rereading with 2D mammography).

The study population was a mix of breast screening assessment cases (85%) and family history screening cases (15%), who were all women at a higher risk of breast cancer by definition. In the TOMMY trial [25], family history screenees were included to provide a group of cases with higher breast density as a result of their lower average age for subanalysis of the impact of breast density on the diagnostic accuracy of DBT, and have therefore been kept in our analysis. Results were essentially unchanged when we excluded the family history screenees, so they apply specifically to women recalled for assessment due to a suspicious screening mammogram. One would expect a smaller effect of density on risk in assessment cases as these would be likely to have higher levels of density on average than the general population, as women with denser breasts have mammography results harder to read and tend to be recalled more often [45]. Our results indicate that the effect in this group, while smaller than observed in the general screened population [2], is by no means negligible. There may be a role for density in the subsequent surveillance and risk management of women recalled for assessment but found not to have breast cancer. Optimisation of the screening technique such as the addition of DBT or increased frequency of screening may be relevant. Alternatively, women could be counselled and offered strategies to reduce their volume of fibroglandular tissue by other lifestyle changes such as stopping hormone replacement therapy, or by primary chemoprevention.

In conclusion, we found that dense breast tissue volume as measured by automated methods was a significant predictor of breast cancer risk in women with a suspicious screening mammogram or a family history. This is consistent with findings that various measures of density can add predictive power to currently used breast cancer risk assessment tools [46], [47]. The fully automated methods can be used with little addition to human resource costs. Density is likely to have a role in risk management both in a population screening context and in management and surveillance of women at increased risk of breast cancer, and in particular can assist in identifying populations who might benefit from enhanced surveillance or primary prevention interventions [47].

## Financial support

The TOMMY trial was supported by a grant from the NIHR Health Technology Assessment Programme.

## Conflict of interest statement

SWD has received research grant funding from Philips. JC has developed educational materials for Hologic. MJM has received research grant funding from Hologic. FJG has received research grant funding from GE Healthcare and payment for lectures from Bracco.

## Role of the funding source

This work was supported by the National Institute for Health Research's Health Technology Assessment Programme [grant number 09/22/182]. The funder had no involvement in the design, conduct, analysis or interpretation.

## Appendix A Supplementary data

mmc1
