# Supplementary material for: Mammographic density and breast cancer risk in breast screening assessment cases and women with a family history of breast cancer
Source: Eur J Cancer. 2018 Jan;88:48–56. doi: 10.1016/j.ejca.2017.10.022 (PMC5768323; doi:10.1016/j.ejca.2017.10.022)
Supplement: mmc1 [file mmc1.docx]

**Mammographic density and breast cancer risk in breast screening assessment cases and women with a family history of breast cancer**

Stephen W. Duffy, Oliver W. E. Morrish, Prue C. Allgood, Richard Black, Maureen G. C. Gillan, Paula Willsher, Julie. Cooke, Karen A. Duncan, Michael J. Michell, Hilary M. Dobson, Roberta Maroni, Yit Y. Lim, Hema N. Purushothaman, Tamara Suaris, Susan M. Astley, Kenneth C. Young, Lorraine Tucker, Fiona J. Gilbert

**Appendix A – Adjustment for total breast volume in place of BMI**

The case for using total breast volume to adjust the effects of percent density measures on risk rests on the correlations of total breast volume and BMI with other breast composition measures and the effects of adjustment in the small subgroup for which we had BMI data. Table A1 shows the correlation matrix of BMI, visual percent density and Volpara breast composition measures. All correlation coefficients were significant at p < 0.001. BMI was negatively correlated with percentage density measures and positively with absolute density measures. Absolute total breast volume was similarly positively correlated with absolute dense volume and negatively correlated with percent density measures. In addition, total breast volume was strongly correlated with BMI (correlation coefficient = 0.71, p < 0.001). The same results were observed when Quantra volumetric measures were used instead of Volpara (data not shown).

The effect of adjustment of effects of percent density measures on risk for Volpara total breast volume was similar to that of adjustment for BMI (restricted only to those with known BMI in both cases, for a fair comparison). The odds ratio per percent visual dense area adjusted for age only was 1.01 (95% CI 0.98-1.03, p = 0.6). When adjusted for BMI, it was 1.03 (95% CI 1.00-1.06, p = 0.02), and when adjusted for Volpara total breast volume, it was 1.03 (95% CI 1.00-1.07, p = 0.01). The odds ratio per Volpara percent dense volume adjusted only for age was 1.00 (95% CI 0.92-1.08, p = 0.9). After adjustment for BMI, the odds ratio was 1.07 (95% CI 0.97-1.17, p=0.1), and after adjustment for Volpara total breast volume, it was 1.08 (95% CI 0.98-1.19, p = 0.1).

We therefore considered it reasonable to adjust for total breast volume in the absence of BMI in the dataset as a whole.

Table A.1. Correlation coefficients among BMI and breast composition measures.

| **Measure** | **BMI** | **Visual % dense area** | **Volpara total breast volume** | **Volpara dense volume** | **Volpara % dense volume** |
| --- | --- | --- | --- | --- | --- |
| **BMI** | 1.00 (–) | – | – | – | – |
| **Visual % dense area** | –0.43 | 1.00 (–) | – | – | – |
| **Volpara total breast volume** | 0.71 | –0.35 | 1.00 (–) | – | – |
| **Volpara dense volume** | 0.53 | 0.16 | 0.61 | 1.00 (–) | – |
| **Volpara % dense volume** | –0.35 | 0.61 | –0.44 | 0.29 | 1.00 (–) |
